# Recurrent gastrointestinal bleeding with ANCA associated glomerulonephritis successfully treated by transarterial embolization

**DOI:** 10.12669/pjms.296.3669

**Published:** 2013

**Authors:** Ling Li, Xiao Li, Ping Fu, Fang Liu

**Affiliations:** 1 Ling Li, Division of Nephrology, West China Hospital of Sichuan University, Chengdu 610041, Sichuan, China.; 2 Xiao Li, Division of Gastroenterology, West China Hospital of Sichuan University, Chengdu 610041, Sichuan, China.; 3 Ping Fu, Division of Nephrology, West China Hospital of Sichuan University, Chengdu 610041, Sichuan, China.; 4 Fang Liu, Division of Nephrology, West China Hospital of Sichuan University, Chengdu 610041, Sichuan, China.

**Keywords:** Anti-neutrophil cytoplasmic antibody-associated vasculitis, Transarterial embolization, Gastrointestinal bleeding

## Abstract

Anti-neutrophil cytoplasmic antibody-associated vasculitis (AAV) is a group of autoimmune diseases that normally affects multiple organs. Recurrent gastrointestinal (GI) bleeding, a critical complication of AAV, remains a challenge. Here, we report a case of AAV complicated by pulmonary hemorrhage, severe recurrent gastrointestinal bleeding, and rapid progressive renal insufficiency that was treated successfully with selective transarterial embolization, continuous veno-venous hemofiltration, plasma exchange, intravenous gamma globulin infusion, followed by steroids and cytotoxic drug therapy. We report this case considering that selective transarterial embolization may be a safe and effective alternative method in recurrent AAV associated GI bleeding caused by AAV refractory to medical therapy.

## INTRODUCTION

Anti-neutrophil cytoplasmic antibody (ANCA)-associated vasculitis (AAV) is a group of autoimmune disease that primarily involves the small blood vessels such as the capillaries vessels, micro-arteries, and venules, and AVV commonly affects multiple organs, in particular the lungs and the kidneys.^1^^-^^3^ To date, recurrent gastrointestinal (GI) bleeding, a critical complication of AAV, is still medical challenge. Here, we present a case with AAV complicated by severe recurrent GI bleeding, followed by rapid progressive renal insufficiency, pulmonary hemorrhage, and pulmonary infection. The patient was treated successfully with selective mesenteric arterial embolization. We report this case considering that selective transarterial embolization may be a safe and effective alternative method in recurrent GI bleeding caused by AAV if uncontrolled by medical therapy.

## CASE REPORT

A 56-year-old man was transferred to nephrology department due to recurrent episodes of fever (38°C～39.2°C), cough, sputum, and thoracalgia for the past three months. The patient had been admitted previously to a local hospital where computer tomography (CT) revealed pleural effusion in both lungs. Laboratory tests showed serum creatinine (sCr) 221.9 μmol/L; proteinuria (3+); and 24 hour proteinuria 5.3g. The patient was diagnosed with chronic renal insufficiency, and pulmonary infection. He was treated with antibiotics for pulmonary infection, but infection-related symptoms gradually worsened and serum creatinine levels reached 434μmol/L within one week. Subsequently, the patient was transferred to our unit for further treatment.

**Fig.1:**
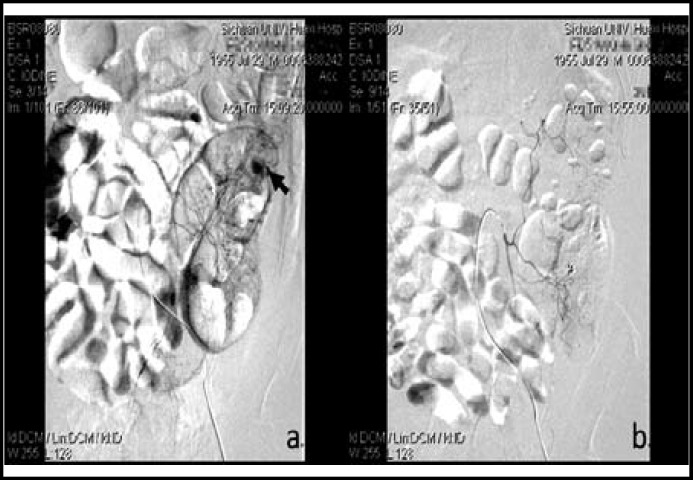
Mesenteric arterial angiography showed a hemorrhagic spot in a branch of the inferior mesenteric artery (a, showed by black arrow). Selective transarterial embolization was performed with coils, and final angiography displayed no signs of arterial bleeding (b).

**Fig.2:**
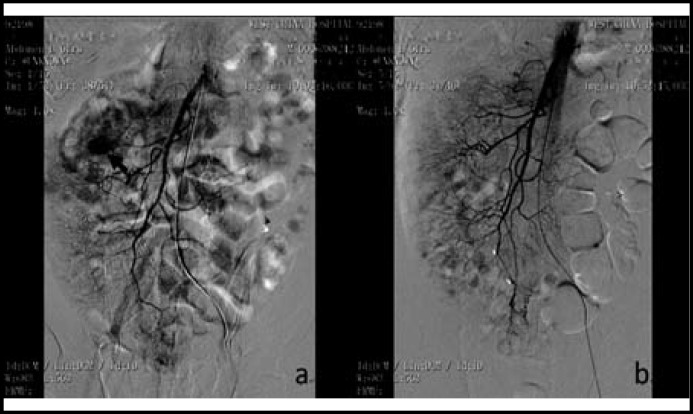
Seven days after the initial procedure, the patient developed GI bleeding symptoms, and mesenteric arterial angiography again showed a hemorrhagic spot in a branch of the inferior mesenteric artery (a, showed by black arrow). Selective transarterial embolization with coils was performed again, and final angiography displayed no signs of arterial bleeding (b).

On admission, physical examination showed T 38°C; R, 18 bpm; HR, 99 bpm; and BP 124/60 mmHg. Laboratory tests found hemoglobin (Hb) 81 g/L; WBC 18.27x10^9^/L, ESR 49.0 mm/H, and sCr 611.0 μmol/L; Urea 22.90 mmol/L; antinuclear antibody (ANA) (−); extractable nucler antigen (ENA) (−); C-reactive protein (CRP) 231mg/L; IgG 10.1g/L; IgA 1650.0 mg/L; IgM 345.0 mg/L; IgE 227.95 IU/mL; RBC 30~40/HPF in the Urine sediment; and 24 hour proteinuria 2.9 g/L. Ultrasound examination of the urologic system showed enlarged kidneys. Additional laboratory tests found P-ANCA (1:10) (+); ANCA-MPO (+); anti-GBM antibody (−). The patient was diagnosed with ANCA-associated vasculitis, AAV associated glomerulonephritis and put on CVVH and antibiotic therapy. After one week, the patient’s infection-related symptoms gradually improved, and the patient was put on intravenous methylprednisolone therapy (80 mg/d).

Four days later, however, the patient developed melana mixed with bloody stool. Laboratory tests revealed Hb 87g/L, Cr 586.9 umol/L, PLT 355×109/L, Fib 2.48 g/L, PT 17.8s, Antithrombin III 92.2%. The gastroscopy and colonoscopy were normal. And capsule endoscopy revealed no obvious abnormalities, including bleeding of mucous membrane. Conventional therapy of GI bleeding (PPI and somatostatin) was ineffective. Finally selective mesenteric arterial angiography was performed, which indicated a hemorrhagic spot in a branch of the inferior mesenteric artery (Fig.1a). Selective transarterial embolization was performed with steel coils (Cook, Bloomington, USA.) (Fig.1b). After the procedure, GI bleeding was completely alleviated. However, two weeks later, the patient presented with hematochezia again and selective transarterial angiography was performed repeatedly, which revealed another hemorrhagic spot in a branch of IMA, and subsequently arterial embolization with coils was performed again (Fig.2a, Fig.2b).

After TAE, the symptoms improved and three days later, the repeated fecal-occult-blood tests were negative, but the vasculitis was not completely controlled for the other symptoms were still existed. The patient was treated with a course of intravenous methylprednisolone pulse therapy (500 mg/d) followed by a course of intravenous cyclophosphamide (CTX) pulse therapy (600mg). Moreover, the patient underwent repeated plasma exchange (8 sections), blood products transfusion, cryoprecipitate therapy, and intravenous gamma globulin infusion (20 g/d for 5 days).

## DISCUSSION

Complications involving the kidneys and the lungs are commonly observed in AAV, of which an entry level serum creatinine and pulmonary hemorrhage are predictors of higher mortality.^4^ In the case of life- or organ-threatening situations, pulse therapy is recommended. In addition, plasma exchange is encouraged if available.^5^^,^^6^

This patient was diagnosed with ANCA-associated vasculitis on the basis of the presence of clinical features and the positive test results for P-ANCA, ANCA-MPO. Renal biopsy was not performed for the bad and severe clinic condition especially bleeding diathesis of this patient.

In our case, during the treatment, the patient presented with hematochezia. As PLT and Fib were within normal ranges and the gastroscopy, colonoscopy and capsule endoscopy revealed no obvious abnormalitie, the DIC and the ulcer bleeding which is caused by the side-effect of corticosteroids is excluded. And lower GI bleeding caused by vasculitis-associated was suspected. This patient had recurrence of GI bleeding for different branch of the mesenteric artery and received the mesenteric arterial angiography and selective transarterial embolization for two times. To our knowledge, the report on AAV that recurred as GI bleeding is rare. As the critical condition of AAV such as life- or organ-threatening situations, the treatment of GI bleeding of AAV is lack of widely accepted method. After the operation the bleeding stopped and there were no postoperative complications such as radiographic contrast nephropathy or mesenteric ischemic necrosis and the patient’s symptom relived.

In conclusion, selective transarterial embolization may be a safe and effective alternative method in recurrent GI bleeding caused by AAV refractory to medical therapy.
